# Synergistic effect on cardiac energetics by targeting the creatine kinase system: in vivo application of high-resolution ^31^P-CMRS in the mouse

**DOI:** 10.1186/s12968-023-00911-6

**Published:** 2023-02-06

**Authors:** Mahon L. Maguire, Debra J. McAndrew, Hannah A. Lake, Philip J. Ostrowski, Sevasti Zervou, Stefan Neubauer, Craig A. Lygate, Jurgen E. Schneider

**Affiliations:** 1 Centre for Preclinical Imaging, University of Liverpool, Liverpool, UK; 2 Division of Cardiovascular Medicine, Radcliffe Department of Medicine, University of Oxford, Oxford, UK; 3 British Heart Foundation Centre for Research Excellence, University of Oxford, Oxford, UK; 4 Experimental and Preclinical Imaging Centre (ePIC), Leeds Institute of Cardiovascular and Metabolic Medicine, University of Leeds, Leeds, UK

**Keywords:** ^31^P-CMRS, Creatine kinase, Creatine transporter, Myocardial function, Transgenic mice, Energetics, Metabolism

## Abstract

**Background:**

Phosphorus cardiovascular magnetic resonance spectroscopy (^31^P-CMRS) has emerged as an important tool for the preclinical assessment of myocardial energetics in vivo. However, the high rate and diminutive size of the mouse heart is a challenge, resulting in low resolution and poor signal-to-noise. Here we describe a refined high-resolution ^31^P-CMRS technique and apply it to a novel double transgenic mouse (dTg) with elevated myocardial creatine and creatine kinase (CK) activity. We hypothesised a synergistic effect to augment energetic status, evidenced by an increase in the ratio of phosphocreatine-to-adenosine-triphosphate (PCr/ATP).

**Methods and results:**

Single transgenic Creatine Transporter overexpressing (CrT-OE, n = 7) and dTg mice (CrT-OE and CK, n = 6) mice were anaesthetised with isoflurane to acquire ^31^P-CMRS measurements of the left ventricle (LV) utilising a two-dimensional (2D), threefold under-sampled density-weighted chemical shift imaging (2D-CSI) sequence, which provided high-resolution data with nominal voxel size of 8.5 µl within 70 min. (^1^H-) cine-CMR data for cardiac function assessment were obtained in the same imaging session. Under a separate examination, mice received invasive haemodynamic assessment, after which tissue was collected for biochemical analysis. Myocardial creatine levels were elevated in all mouse hearts, but only dTg exhibited significantly elevated CK activity, resulting in a 51% higher PCr/ATP ratio in heart (3.01 ± 0.96 vs. 2.04 ± 0.57—mean ± SD; dTg vs. CrT-OE), that was absent from adjacent skeletal muscle. No significant differences were observed for any parameters of LV structure and function, confirming that augmentation of CK activity does not have unforeseen consequences for the heart.

**Conclusions:**

We have developed an improved ^31^P-CMRS methodology for the in vivo assessment of energetics in the murine heart which enabled high-resolution imaging within acceptable scan times. Mice over-expressing both creatine and CK in the heart exhibited a synergistic elevation in PCr/ATP that can now be tested for therapeutic potential in models of chronic heart failure.

## Introduction

The myocardial creatine kinase (CK) system has an important role in maintaining energy provision in the heart [1]. The mitochondrial isoform of CK (CK-Mt) catalyses the transfer of a phosphoryl moiety from adenosine triphosphate (ATP) onto creatine to form phosphocreatine (PCr), which accumulates to high levels in cardiomyocytes and acts to buffer against highly fluctuating energy demands. The cytosolic muscle isoform of CK (CK-M) catalyses the reverse reaction to rapidly regenerate ATP at sites of utilisation, generating free creatine to complete the cycle. In this way, the CK system does not require ATP diffusion and thereby maintains thermodynamically favourable local reactant concentrations [2].

It has long been recognised that impairment of the CK system is a fundamental feature of the chronically failing heart [3]. For example, lower levels of myocardial creatine were first described in the 1930’s [4], while CK isoenzyme activity is also reduced regardless of heart failure aetiology [1, 3, 5]. However, these observations are based on post-mortem analyses and do not therefore reflect the high workloads in vivo, when the CK system is thought to be most critical [6]. The advent of phosphorus cardiovascular magnetic resonance spectroscopy (^31^P-CMRS) has allowed the measurement of high energy phosphates in the failing human heart, typically expressed as the ratio of phosphocreatine (PCr)/ATP, since ATP levels are relatively stable and obtaining absolute values is technically challenging [7]. In this way, a reduction in PCr/ATP has been demonstrated in human dilated cardiomyopathy, which positively correlates with left ventricular (LV) ejection fraction and represents an independent biomarker of disease severity and outcome [8–10].

The application of in vivo ^31^P-CMRS to murine models of chronic heart failure has also provided important insights. For example, transgenic mice overexpressing CK-M in the heart maintained higher levels of PCr/ATP following induction of pressure-overload heart failure and this was associated with improved contractility and survival [11]. However, the small scale and high heart rate of the mouse makes cardiac ^31^P-CMRS intrinsically difficult, which has typically required methodological compromise and limited the application to relatively few groups worldwide.

Previous ^31^P-CMRS studies have employed either single voxel CMRS or chemical shift imaging (CSI) approaches to measure PCr and ATP in the beating mouse heart. CSI-based approaches include 1D CSI measurements, which used phase encoding to localize voxel depth and the limited size of the CMR surface coil placed adjacent to the heart to restrict the in-plane field of view [12–14]. Such an approach is substantially reliant on the surface coil sensitivity profile in order to avoid significant contamination of the signal from the proximal wall of the myocardium with signal from both the chest wall and blood in the ventricular lumen. As the CSI voxels were planar whilst both the chest wall and myocardium are curved, this localisation approach represents an approximation. Two-dimensional (2D) CSI has been employed and offers voxel shapes that better approximate the geometry of the myocardium [15]. By acquiring data in the short-axis orientation, the cuboidal voxels of the CSI point approximately along the midventricular wall of the myocardium making partial volume contamination of the signal more predictable. Acquisition weighting was also employed in order to increase the signal-to-noise ratio (SNR) at the expense of spatial resolution. However, due to the low resolution of the acquired data, the resulting voxel volumes were large and substantial signal contamination from outside the voxel of interest can be expected. More recent studies have successfully used Image Selected In vivo Spectroscopy (ISIS) to acquire ^31^P spectra from a single voxel placed around the entire LV [16]. An actively decoupled volume coil with small surface coil were used to increase sensitivity resulting in excellent in vivo cardiac ^31^P spectra. The relatively long repetition time (TR) required for ISIS means that the data acquisition process is slow and the spatial localization employed is prone to motion artefacts if care is not taken. The T_1_ saturation of the metabolite amplitudes was compensated for by comparing with those from fully relaxed spectra acquired in a subset of animals. Ex vivo measurements of large volume blood samples made using the same imaging protocol were used to correct for blood contamination of the ATP resonances in the heart. It was found that the blood signal contributed little to the overall ATP signal observed in the heart although the resulting PCr/ATP ratios reported were low when compared to the existing literature. It remains unclear though whether this was due to degradation of the blood sample during the acquisition of the ex vivo spectra.

The purpose of this study was to improve on available ^31^P-CMRS methodologies for assessing cardiac energetics in the murine heart by utilising an optimised volume-coil-transmit and surface coil array receiver radiofrequency (RF) coil setup and implementing a slice-selective, density-weighted CSI technique that allows for high-resolution data acquisition (nominal voxel size: 8.5 µl) within ~ 70 min. We apply this to a novel double transgenic mouse model (dTg), which over-expresses both CK-M and the creatine transporter (CrT) in the heart. We have previously shown that CrT overexpressing mice (CrT-OE) have elevated levels of total creatine and phosphocreatine in the heart, but that the relative proportion of PCr is limited by endogenous CK activity [17]. We hypothesise that enriching both substrate and enzyme will have a synergistic effect on PCr/ATP ratio. Specifically, we sought to establish whether compared to the CrT-OE single transgenic (i) the dTg heart does indeed have higher PCr/ATP ratio; (ii) adding a second transgene does not have unforeseen impacts on LV function over-and-above those previously described [17]. A positive outcome would suggest the utility of this approach for treatment of chronic heart failure.

## Methods

### Ethics statement and animal husbandry

All animal experiments were approved under project licence 30-3314 by the Committee for Animal Care and Ethical Review at the University of Oxford and comply with the UK Animals (Scientific Procedures) Act 1986, as amended 2012. CrT-OE mice were from the Tg55 line first described by Wallis et al. (2005), which over-express rabbit CrT (SLC6A8) under control of the cardiac-specific murine ventricular myosin light chain 2 (MLC2v) promoter [17]. Cardiac-specific MCK-OE mice, Tg(*Myh6-CKm)-6*, were generated in our core transgenic facility as described below. Both mouse lines were maintained by breeding heterozygotes with C57BL/6J^OlaHsd^ wildtypes (Envigo, Huntingdon, UK) and have been backcrossed for > 10 generations. Heterozygotes from each line were used to generate dTg and littermate controls. Only males were used in this study since transgenic offspring from CrT-OE mice are predominately male.

Mice were group housed in individually ventilated cages under specific pathogen-free conditions at 21 °C and controlled humidity (50–55%) with a 12-h light–dark cycle. Water and chow were available ad libitum using irradiated 2016 Teklad Global 16% Protein Rodent Diet, which is naturally creatine-free (Envigo, Huntingdon, UK).

### Generation of CKM-OE mice and genotyping

The transgenic construct α-myocin heavy chain (MHC)-CKM containing the mouse Myh6 (α-MHC) promoter driving the expression of the murine Ckm cDNA cassette, was injected into the pronucleus of fertilized oocytes obtained from B6CBAF1 females. Following overnight cultivation, the surviving two-cell embryos were transplanted into pseudopregnant CD1 foster mothers at 0.5 days post-coitum. The resulting pups were genotyped and two independent transgenic founder mice were identified by polymerase chain reaction (PCR). The strain reported here has random integration of 6 copies of the transgene and has been backcrossed with C57BL/6J^OlaHsd^ mice until congenic. Animals were genotyped at weaning from an ear biopsy using PCR under standard conditions (CrT forward, 5′-GCATCTTCATCTTCAACATCGTGTA-3′ and reverse, 5′-TCACAGATCCTCTTCTGAGATGAG-3′; CKM forward 5′-GCACAGGTGGCGTGGACA-3′ and reverse, 5′-TGCGTAATCTGGAACATCGT-3′). Breeding produced Mendelian ratios of transgenic offspring.

### Experimental protocol

This study was performed in n = 7 CrT-OE and n = 6 dTg male mice, which were subjected to cardiac cine CMR examination followed by in vivo ^31^P-CMRS (mean age 22 ± 3 weeks). Invasive LV haemodynamics were measured at 31 ± 3 weeks, after which cardiac tissue was snap frozen for biochemical measurements.

### In vivo^31^P-CMRS

**Fig. 1:**
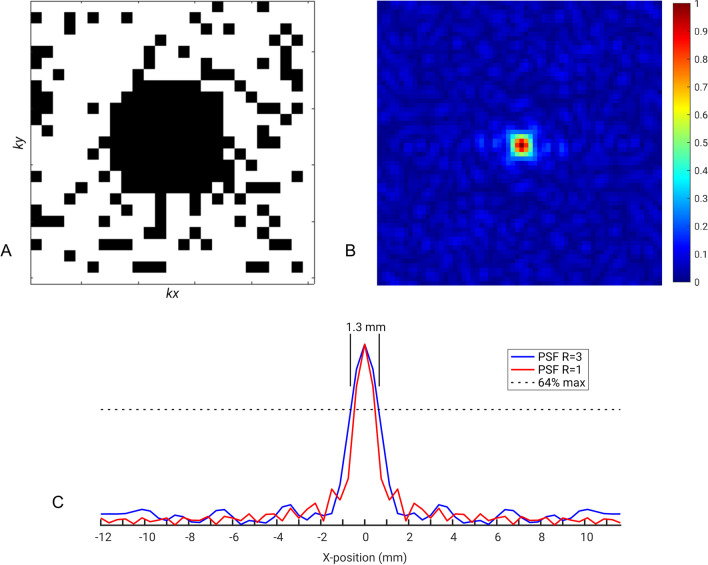
Density-weighted three-fold under-sampling of the acquired k-space data was used. The data acquisition mask used to acquire the data is shown (**A**). The resulting point-spread function (PSF) is shown after zerofilling to a matrix size of 64 × 64 and FOV of 24 × 24 mm^2^ (**B**) along with the corresponding profile through the centre of the three-fold under-sampled (R = 3) PSF (**C**). Quantitative analysis of the PSF at 64% maximum peak height yielded a nominal spatial resolution of ~ 1.3 mm. The corresponding PSF resulting from an equivalent uniformly sampled acquisition (R = 1) is shown for comparison

Mice were anaesthetised with 4% isoflurane in 100% medical oxygen and maintained on a nose cone at 1.5–2% throughout. Mice were placed prone on a thermo-regulated cradle with electrocardiogram (ECG) and respiratory gating. Data were acquired on a 9.4 T CMR system comprising of a DirectDrive2 console, a 120 mm i.d., 0.6 T/m, shielded gradient set (Agilent Technologies, USA), a linear double-tuned, actively decoupled ^1^H/^31^P birdcage resonator (i.d. 39 mm) and a 14 mm actively decoupled 2-element surface coil array with quadrature-combiner for ^31^P signal reception (Rapid Biomedical, Germany) as previously described [18]. In brief, a two-dimensional, slice-selective, threefold undersampled density-weighted CSI (2D-CSI) sequence was used in short-axis orientation to acquire spectroscopic measurements in vivo (field-of-view (FOV) 24 × 24 mm^2^, 24 × 24 phase encoding (PE) steps, 5 mm mid-ventricular slice, 16,296 free induction decays (FIDs), 30° flip angle, bandwidth 8013 kHz, 1024 complex points, cardiac triggered, TR ≈ 250 ms [i.e. two cardiac cycles], total acquisition time ~ 67 min). The sampling pattern is shown in Fig. 1A, and a surface plot of the resulting spatial response function in Fig. 1B. Prior to Fourier transform, the data were zero-filled to 64 × 64 PE steps to improve the apparent spatial resolution of the images. Quantitative analysis of the spatial response function (Fig. 1C) at 64% of maximum peak height [19] yielded a nominal in-plane resolution of 1.3 × 1.3 mm^2^, resulting in a voxel volume of ~ 8.45 µl. A line broadening of 60 Hz was applied to improve the SNR of the resulting spectra.

The data were reconstructed using IDL 8.2 (Harris Geospatial Solutions, L3Harris Geospatial, Boulder, Colorado, USA) and spectra corresponding to voxels placed in the myocardium and blood were fitted in the time domain using in-house software [20]. A voxel placed in the lumen of the LV was used to quantify the [2, 3]-DPG/γ-ATP in the blood and correct the myocardial ATP levels for signal contamination from blood. Correction of T_1_ saturation effects was carried out in Excel 2014 (Microsoft Corporation, Redmond, Washington, USA), and T_1_ values for PCr and ATP in the mouse myocardium at 9.4 T were taken from the literature [15].

### Left ventricular function

LV cardiac function was obtained in the same imaging session. Eight to ten contiguous, 1-mm-thick slices were then acquired in short-axis orientation covering the entire heart using an ECG-triggered and respiratory gated multi-frame, two-fold undersampled compressed-sensing accelerated [21] sequence with steady-state maintenance during respiration [22]. The imaging parameters were: FOV 30 × 30 mm^2^, matrix size 128 × 128, TE/TR = 1.7/4.6 ms, 15° sinc excitation pulse, number of averages NT = 8. The number of frames per cardiac cycle was determined by the heart rate. Data were analysed using the three-dimensional guide-point modelling as reported previously [23, 24].

### Haemodynamics

For haemodynamic assessment, the LV was cannulated via the right carotid artery with a 1.4F Millar pressure catheter (SPR-839, Millar Instruments, Houston, TexasX, USA). Mice were given 15 min equilibration before measurements were taken under baseline and stimulated conditions using a Powerlab 4SP (ADInstruments, Castle Hill, Australia). Mice were then killed by intravenous overdose of pentobarbitone.

### Biochemistry

Hearts were excised, rinsed with saline, blotted on tissue, LV and right ventricle (RV) dissected, then rapidly frozen using Wollenberger tongs chilled in liquid nitrogen. Samples were stored at − 80 °C until utilised. Frozen LV tissue was powdered for quantification of total creatine levels by HPLC using the method of Teerlink et al*.* [25] and normalised to protein content using the Lowry method. Total CK activity was quantified spectrophotometrically under saturating conditions as previously described [26]. CK isoenzymes were separated according to their electrophoretic mobility on an agarose gel. Relative activities of individual CK isoenzymes were quantified by densitometry using a SAS-1 CK VIS-12 Isoenzyme kit (Helena Biosciences, Gateshead, UK). Citrate synthase activity was measured spectrophotometrically as previously described [27].

### Statistics

Each data set was analysed by a single operator who was blinded to genotype. Data are presented as mean ± standard deviation and all statistical tests were calculated using GraphPad Prism (version 9.1.2 for Windows; GraphPad Software, San Diego, CA, USA). An unpaired Student’s t-test was used for comparison of means between two groups. An F-test was applied to compare variances and an Anderson–Darling test to check for normality of residuals.

## Results

**Fig. 2:**
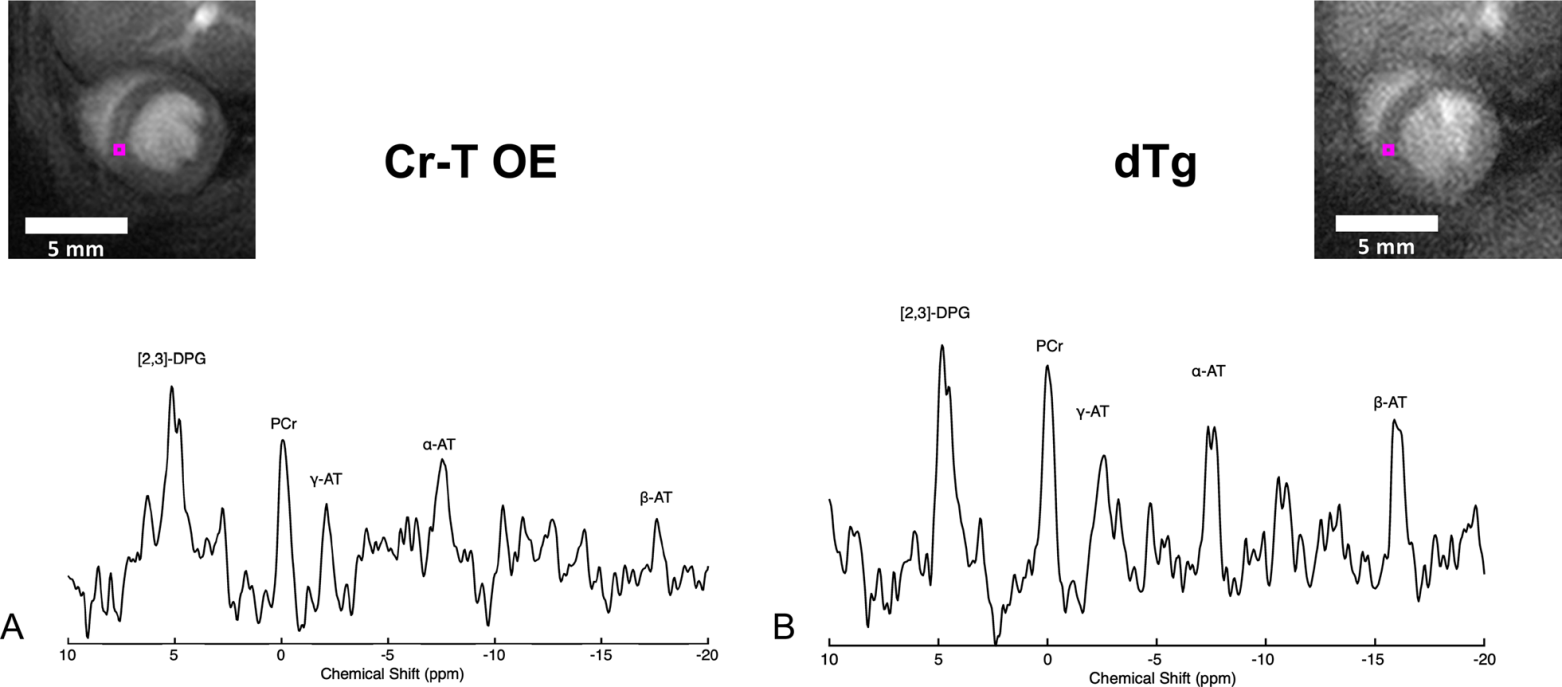
Representative spectra from a voxel placed in the left ventricular septal wall of (**A**) a creatine transporter overexpressing mouse and (**B**) a double transgenic mouse, with corresponding short-axis scout images showing voxel position (magenta square). Spectra show peaks for 2,3-diphosphoglycerate ([2, 3]-DPG), phosphocreatine (PCr) and the γ, α, and β phosphoryl groups of adenosine-triphosphate (ATP). A 5 mm scalebar is shown for reference

We generated a new model of constitutive CK-M overexpression specifically in the heart and crossed this with a pre-existing line overexpressing the CrT (CrT-OE) to make dTg animals. All comparisons are between single transgenic CrT-OE and the dTg mice. Figure 2 shows representative ^31^P-CMRS spectra from voxels in the septal wall of (A) a CrT-OE and (B) a dTg mouse respectively, with the voxel locations indicated on the anatomical ^1^H-images. PCr and α-, β-, and γ-ATP resonances can clearly be seen, with the [2, 3]-DPG resonance arising from signal contamination from the blood.

**Fig. 3:**
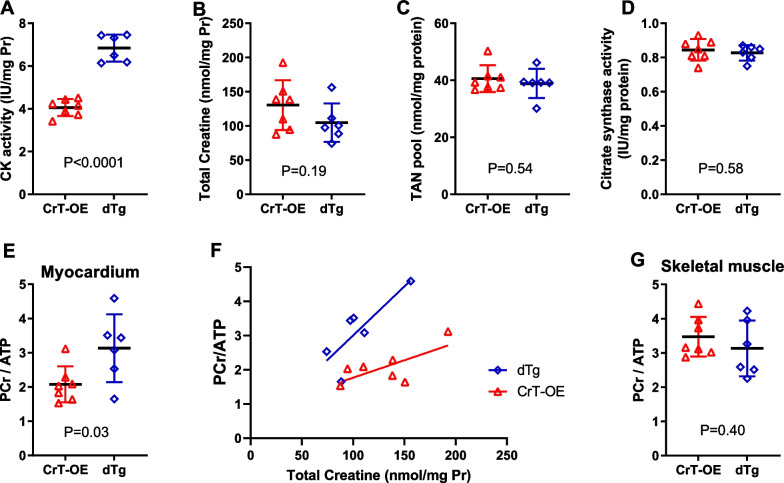
Left ventricular biochemistry in creatine transporter overexpressing mice (CrT-OE, n = 7) and in double transgenic animals (dTg, n = 6). Total creatine kinase (CK) activity was significantly elevated in dTg hearts (**A**), while there were no significant differences in total creatine levels (**B**), total adenine nucleotides (**C**), or in citrate synthase activity (**D**). In vivo ^31^P-CMRS found significantly higher PCr/ATP ratio in dTg hearts (**E**) and a linear relationship with total creatine levels (CrT-OE: y = 0.01018*x + 0.7489, r^2^ = 0.49; dTg: y = 0.02864*x + 0.1378, r^2^ = 0.66) with a significantly different intercept (P = 0.002), indicating that PCr/ATP levels are higher in dTg for any given value of total creatine (**F**). In contrast, there was no difference in PCr/ATP levels in the skeletal muscle adjacent to the heart (**G**). Data are mean ± SD with comparisons by unpaired Student’s t-test

We have previously shown that CK activity and isoenzyme distribution is unaltered in CrT-OE compared to wild-type littermates [17]. However, hearts from dTg mice had 69% higher levels of total CK activity (Fig. 3A). The cytosolic muscle (M) and brain (B) CK isoforms can form homodimers or heterodimers and the relative distribution is shown in Table 1. Activity of the high abundance CK-MM isoenzyme was 2.25-fold higher in dTg hearts and the lower abundance CK-MB was threefold higher, validating that specific protein activity of the muscle-type isoform of CK, i.e. CK-M, was indeed elevated in dTg animals, while CK-BB was near the limit of detection. There was an unexpected 31% reduction in CK-Mt activity suggesting an element of functional reciprocity between these isoenzymes.

**Table 1:** Creatine kinase isoenzyme activity in the left ventricle of creatine transporter (CrT) overexpressing and double transgenic (dTg) mice

	CrT-OE (n = 7)	dTg (n = 6)	P value
CK-Mt	1.53 ± 0.15	1.09 ± 0.51	0.09
CK-MM	2.02 ± 0.35	4.48 ± 0.98	0.001
CK-MB	0.40 ± 0.08	1.18 ± 0.31	0.001
CK-BB	0.09 ± 0.02	0.09 ± 0.03	0.65

Data are mean enzymatic activity (IU/mg protein) ± SD with comparisons by unpaired Student’s t-test

*CK-BB* brain-type creatine kinase, *CK-MM* muscle-type creatine kinase, *CK-MB* muscle and brain creatine kinase

Both groups had levels of myocardial total creatine (free creatine + PCr) > 100 nmol/mg protein (Fig. 3B), which is elevated compared to historical wild-type values (66 ± 6 nmol/mg protein) and showed a wide distribution as previously reported [17]. This did not affect levels of total adenine nucleotides (AMP + ADP + ATP) or citrate synthase activity, which is a marker for mitochondrial cell density (Fig. 3C, D). Using in vivo ^31^P-CMRS, the ratio of PCr/ATP was found to be 51% higher in dTg hearts compared to CrT-OE (i.e. 3.01 ± 0.96 vs. 2.04 ± 0.57—mean ± SD; dTg vs. CrT-OE) and was significantly correlated with total creatine values in the dTg only (r = 0.81; P = 0.04). There was a significant difference between the elevations in these relationships (P < 0.002) indicating that for any given value of creatine, the dTg hearts contained more PCr (Fig. 3E, F). PCr/ATP was also measured in skeletal muscle from the chest wall as an internal control and was found to be no different between genotypes as expected (Fig. 3G). The F-test for comparing variances of myocardial PCr/ATP yielded no significant difference (P = 0.16) and the residuals were normally distributed (P = 0.54).

**Fig. 4:**
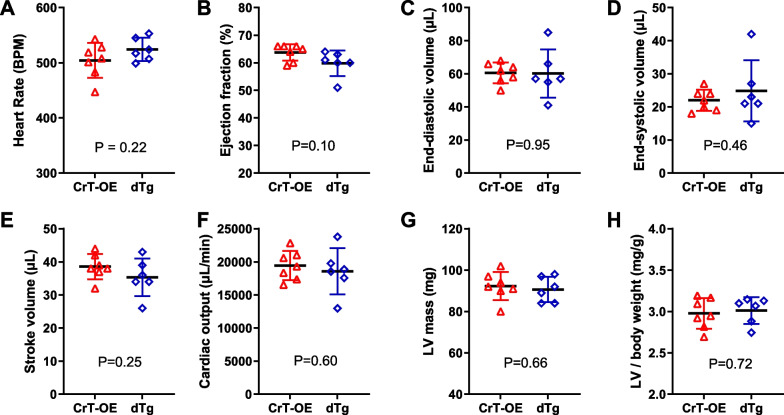
Left ventricular morphology and function. Cine-CMR of the LV in creatine transporter overexpressing mice (CrT-OE, n = 7) and in double transgenic animals (dTg, n = 6). **A** Heart rate, **B** ejection fraction, **C** end-diastolic volume, **D** end-systolic volume, **E** stroke volume, **F** cardiac output, **G** LV mass derived from CMRI, **H** LV mass/body weight ratio. Data are mean ± SD and no significant differences were observed between groups for any parameter using an unpaired Student’s t-test

We have previously found that very high levels of creatine (> twofold) is associated with LV hypertrophy and dysfunction in the CrT-OE mouse [17, 28], and therefore measured baseline cardiac function to determine if there were any unexpected consequences of also overexpressing CK-M. Creatine levels in these cohorts were not within the harmful range; impairment of function was not evident, nor was there any significant effect of CK-M over-expression on LV function as measured by CMR (Fig. 4) or invasive haemodynamics (Table 2). Therefore, the augmentation of both creatine and CK activity in the heart did not result in any measurable effect on cardiac structure and function when compared to elevated creatine alone.

**Table 2:** In vivo left ventricular (LV) haemodynamics in CrT overexpressing (CrT-OE) and double transgenic (dTg) mice

	CrT-OE (n = 7)	dTg (n = 6)	P value
Heart rate (BPM)	377 ± 68	405 ± 67	0.47
LV end-systolic pressure (mmHg)	100 ± 6	99 ± 8	0.78
LV end-diastolic pressure (mmHg)	6.4 ± 3.4	6.4 ± 3.1	0.99
dP/dt_max_ (mmHg/s)	5929 ± 1376	6162 ± 1862	0.80
dP/dt_min_ (mmHg/s)	− 5639 ± 1401	− 6070 ± 2424	0.70
Tau of isovolumetric relaxation (ms)	9.6 ± 1.4	10.3 ± 3.4	0.62

Data are mean ± SD with comparisons by unpaired Student’s t-test

## Discussion

We have reported on the implementation of a ^31^P metabolic imaging technique that provides the highest resolution reported to date (voxel volume: 8.5 µl), which is > 3.5 × smaller than in our previous study [18]. The use of an actively decoupled volume coil in conjunction with a small surface coil array has yielded sufficient SNR in the proximal wall and septum of the heart to enable quantification of PCr/ATP ratios even with such low voxel volumes. The application of a density weighted acquisition scheme also enabled an improvement in the achievable SNR with only a modest cost in terms of spatial resolution when compared to an equivalent uniformly sampled acquisition. These features of our methodology enable acquisition of high-resolution data in a physiological reasonable timescale (~ 70 min) but necessitated the design of a different density weighted undersampling pattern than in [18]. With this acquisition time for the CSI data, and as the volume coil was double resonant, functional imaging could also be acquired in the same imaging session, thereby enabling direct correlation of cardiac energetics with LV functional readouts.

The short TR used for the ^31^P data acquisition resulted in substantial T_1_ saturation of the resonances in the spectra. This required correction of the PCr and ATP signal intensities resulting from the fitting of the spectra to obtain accurate PCr/ATP ratios in the myocardium. Signal contamination from the blood arising from partial volume effects, as evidenced by the presence of a [2, 3]-DPG resonance in the ‘myocardial’ spectrum, results in the blood contributing substantially to the apparent ATP concentration in the voxel. As we have used CSI, a voxel placed in the lumen of the LV can be used to quantify the [2, 3]-DPG/γ-ATP in the blood and correct the myocardial ATP levels prior to correction for T_1_ saturation. Since both the myocardial and blood spectra were simultaneously acquired in the same animal, no correction for T_1_ of blood resonances is therefore required. Similarly, a reference spectrum from an ex vivo blood sample is not required therefore eliminating the need to account for ex vivo sample degradation or for inter-animal variation.

The CK system in our experimental groups, which were matched for age (i.e. 22 weeks ± 3 weeks), is fully developed and myocardial creatine levels can therefore be expected to be static in the mature animal [28, 29].

An inducible mouse model of CK-M-OE has previously been described by Gupta et al*.*, who did not measure CK-M activity directly, but described a ~ 70% higher levels for both CK-M protein expression and total CK activity. Herein, we observed a similar 69% increase in total CK activity, driven by a 2.2-fold increase in CK-M-specific activity. Although this was measured in dTg mice, we did not previously observe changes in CK activity in the CrT-OE model [17]. This suggests that our CK-M-OE has comparable CK activity to the published model, which was found to be beneficial in multiple disease states, e.g. pressure-overload heart failure [11], ischaemia/reperfusion injury [30] and doxorubicin cardiotoxicity [31]. Notably, Gupta et al. measured PCr and ATP concentrations and CK flux with CMRS, but not CK isoenzyme activity as done biochemically in the present study [31].

Over-expression of the CrT results in accumulation of myocardial creatine, which, within a therapeutic range of 1.2 to twofold above normal values, has been shown to be cardioprotective in models of ischaemia/reperfusion injury [32, 33]. However, total creatine above twofold was associated with LV hypertrophy and impaired contractility [17, 28]. This may reflect feedback mechanisms related to PCr and Cr concentrations directly, or an inability to maintain sufficient creatine in the phosphorylated state (i.e. phosphocreatine), which negatively impacts on the free energy available from ATP hydrolysis (ΔG_ATP_) [17]. This suggests that the expanded creatine pool exceeds the limits of normal CK capacity, and therefore, that simultaneous overexpression of CK may correct this deficit, thereby maintaining even higher levels of total creatine and PCr without deleterious effects on the heart. The dTg mice reported herein were created in order to test this hypothesis, and our finding that PCr/ATP ratio is increased compared to single transgenic CrT-OE validates this approach (3.01 ± 0.96 vs. 2.04 ± 0.57). For reference, previous work has measured PCr/ATP in wild type animals to be 1.68 ± 0.64 [18]. Future studies will screen larger numbers of mice to identify individuals with creatine levels within the harmful (> twofold range) to determine whether increased CK activity maintains normal cardiac function. If so, this would make available a wider therapeutic range for elevated creatine, which can then be tested in disease models of acute myocardial infarction and chronic heart failure.

In conclusion, we report on a refined high-resolution ^31^P imaging technique suitable for the in vivo assessment of high energy phosphates in mouse heart. Application of this technology to a novel mouse model with increased levels of both creatine and creatine kinase activity demonstrated a synergistic increase in PCr/ATP ratio. Future proof-of-principle studies will test the therapeutic potential of this approach in chronic heart failure, with high-resolution ^31^P-CMRS providing mechanistic insight via assessment of cardiac energetics.

## Abbreviations

[2, 3]-DPG: 2,3-Diphosphoglyceric acid
^31^P-CMRS: Phosphorus cardiovascular magnetic resonance spectroscopy
ATP: Adenosine triphosphate
BWt: Body weight
CK: Creatine kinase
CK-BB: Brain-type creatine kinase (homodimer)
CK-M: Muscle-type isoform of creatine kinase
CK-MM: Muscle-type creatine kinase (homodimer)
CK-MB: Muscle and brain creatine kinase (heterodimer)
CK-Mt: Mitochondrial isoform of creatine kinase
CMRS: Cardiovascular magnetic resonance spectroscopy
CrT: Creatine transporter
CrT-OE: CrT overexpressing mice
CSI: Chemical shift imaging
dTg: Double transgenic
ECG: Electrocardiogram
FID: Free induction decay
FOV: Field-of-view
HPLC: High-performance liquid chromatography
i.d.: Inner diameter
ISIS: Image selected in-vivo spectroscopy
LV: Left ventricle/left ventricular
MHC: Myosin heavy chain
NT: Number of transients (averages)
PCr: Phosphocreatine
PCR: Polymerase chain reaction
PE: Phase encoding
PSF: Point spread function
RF: Radiofrequency
RV: Right ventricle/right ventricular
SNR: Signal-to-noise ratio
TE: Echo time
TR: Repetition time

## Declarations

### Availability of data and materials

The datasets used and analysed during the current study are available from the corresponding author on reasonable request.
